# A direct repeat of E-box-like elements is required for cell-autonomous circadian rhythm of clock genes

**DOI:** 10.1186/1471-2199-9-1

**Published:** 2008-01-04

**Authors:** Yasukazu Nakahata, Mayumi Yoshida, Atsuko Takano, Haruhiko Soma, Takuro Yamamoto, Akio Yasuda, Toru Nakatsu, Toru Takumi

**Affiliations:** 1 Osaka Bioscience Institute, Suita, Osaka 565-0874, Japan; 2 Kyoto University Graduate School of Biostudies, Sakyo, Kyoto 606-8501, Japan; 3 Life Science Laboratory, Material Laboratories, Sony Corporation, Shinagawa, Tokyo 144-0001, Japan; 4 Department of Structural Biology, Graduate School of Pharmaceutical Sciences, Kyoto University, Sakyo, Kyoto 606-8501, Japan; 5 Department of Molecular Neuroscience, Kyoto University Graduate School of Medicine, Sakyo, Kyoto 606-8501, Japan

## Abstract

**Background:**

The circadian expression of the mammalian clock genes is based on transcriptional feedback loops. Two basic helix-loop-helix (bHLH) PAS (for Period-Arnt-Sim) domain-containing transcriptional activators, CLOCK and BMAL1, are known to regulate gene expression by interacting with a promoter element termed the E-box (CACGTG). The non-canonical E-boxes or E-box-like sequences have also been reported to be necessary for circadian oscillation.

**Results:**

We report a new cis-element required for cell-autonomous circadian transcription of clock genes. This new element consists of a canonical E-box or a non-canonical E-box and an E-box-like sequence in tandem with the latter with a short interval, 6 base pairs, between them. We demonstrate that both E-box or E-box-like sequences are needed to generate cell-autonomous oscillation. We also verify that the spacing nucleotides with constant length between these 2 E-elements are crucial for robust oscillation. Furthermore, by *in silico *analysis we conclude that several clock and clock-controlled genes possess a direct repeat of the E-box-like elements in their promoter region.

**Conclusion:**

We propose a novel possible mechanism regulated by double E-box-like elements, not to a single E-box, for circadian transcriptional oscillation. The direct repeat of the E-box-like elements identified in this study is the minimal required element for the generation of cell-autonomous transcriptional oscillation of clock and clock-controlled genes.

## Background

Circadian rhythms, periodicities with an approximate 24-h length, are essential physiological functions in almost all organisms on the earth and are generated by endogenous biological clocks. The biological clocks consist of 3 components: input, pacemaker, and output. The endogenous period length is not exactly 24 h and must be entrained by light. In mammals, other entrainment cues such as food intake or temperature, even social cues, can reset the circadian rhythm. The central oscillator in mammals is located in the suprachiasmatic nuclei (SCN) in the anteroventral hypothalamus. Recent studies revealed that the peripheral clocks reside not only in other brain regions but also in peripheral organs and even in cultured cells [1-4]. The output of the biological clocks includes broad physiological phenomena such as locomotor activity, sleep-wake cycle, hormonal secretion, cardiovascular condition, bowel movement, and even mental states. The disturbance of the biological clocks may cause many dysfunctions from routinely experienced jet-lag or work-shift problems to sleep disturbance and cardiovascular, metabolic or mental diseases [2,5].

Recent molecular biological studies have revealed that the molecular mechanism of the biological clocks is based on the interlocked loops of transcriptional and translational feedback [6-8]. Among the transcriptional controls in mammals, 3 major clock components for canonical clock genes, i.e., E-box, RORE, and DBPE, have been reported so far [3,9]. The E-box (CACGTG), for example, in the *Per1 *promoter, is the most well-known regulatory element. It is considered the binding site for the heterocomplex of CLOCK/BMAL1, which is a positive regulatory element. Negative regulatory elements, PERs and CRYs, restrain the transcriptional activity [10-12]. It is widely known that not only other clock genes are regulated by the E-box but also output regulators or clock-controlled genes are regulated by it as well [13-17]. On the other hand, *Bmal1*, whose circadian expression exhibits a sort of anti-phase to *Per2 *expression, is positively regulated by *Rorα *and negatively regulated by *Rev-erbα*, through RORE [18-21].

*Per2 *was literally cloned as a second mammalian *period *gene [22,23], however, gene-knockout analysis revealed a more prominent role for mPER2 in the mammalian clock than PER1 [24]. Using the *in vitro *rhythm oscillation monitoring system (IV-ROMS) [3,25], instead of the canonical E-box (CACGTG), we previously identified an E-box-like sequence (CACGTT) in the *Per2 *promoter region *in vitro *[26], which is essential for the cell-autonomous transcriptional oscillation of *Per2 *as well as for *Per2 *circadian oscillations *in vivo *[27]. For the cell-autonomous transcriptional oscillation of *Per2*, not only this E-box-like sequence but also the downstream region is involved in the circadian oscillation of *Per2 *[26]. Here, we identified closely spaced E-box or E-box-like elements in the regulatory regions of canonical clock genes including *Per2*. Our comprehensive approach raised the possibility that not just a single E-box but a direct repeat of E-box-like elements is required for circadian oscillation of core clock and clock-controlled genes.

## Results

**Figure 1:**
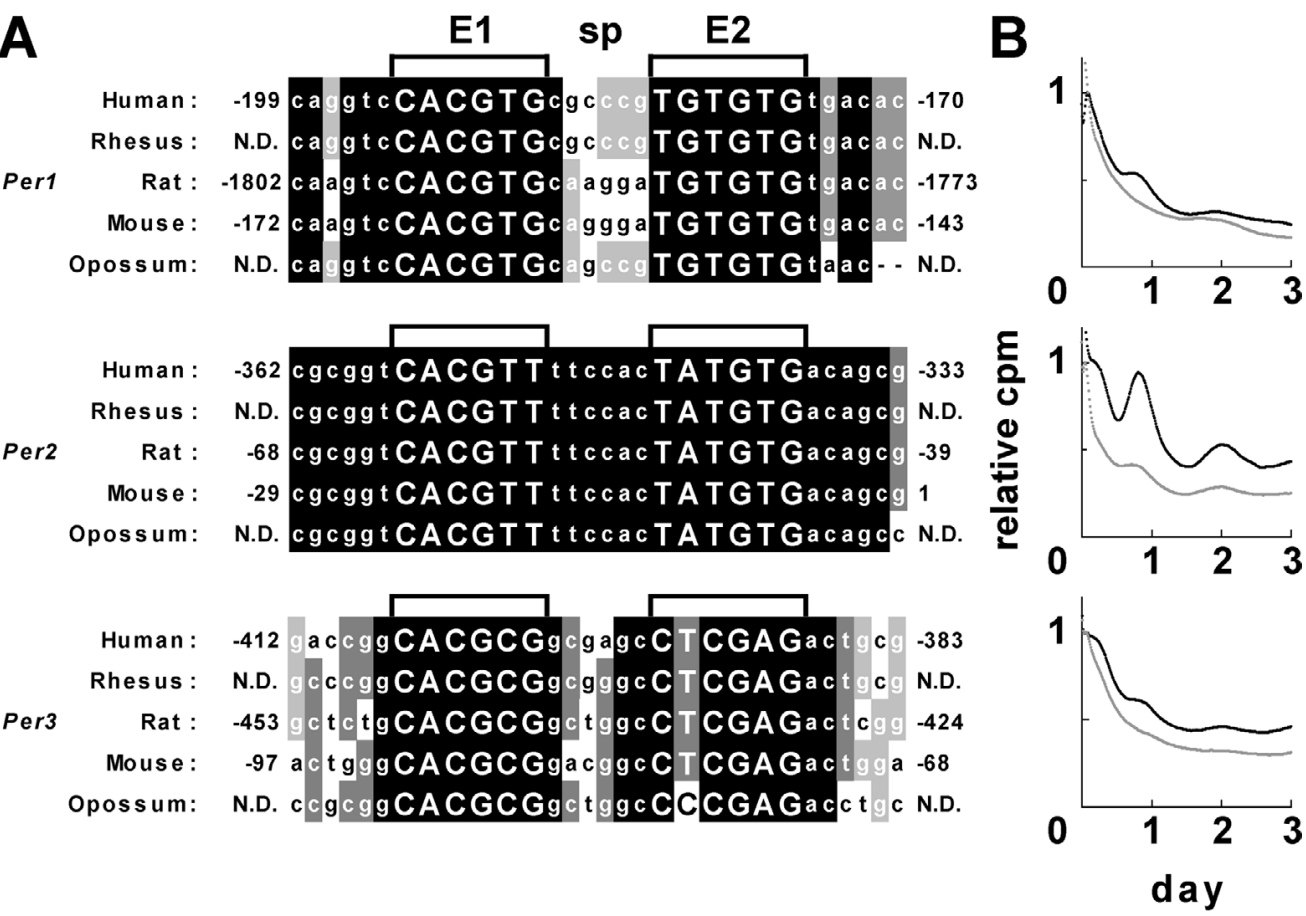
**The "EE-element" possesses transcriptional activity displaying a circadian rhythm**. (A) Sequence alignments of the promoter region, containing each EE-element of *Per1, Per2 and Per3 *gene, among human, rhesus, rat, mouse and opossum (The NCBI accession numbers are indicated in Additional file 6). The core sequences (E1 and E2) within the EE-element are shown in capital letters. Numbers at both sides of alignments indicate the position from the transcription start site (TSS). (B) EE-element-driven luciferase bioluminescence by IV-ROMS. Black lines indicate bioluminescence after serum shock; and gray lines show the negative control (replacement with same medium). Abscissa presents "day"; and ordinate, "relative luciferase intensity". First peak values of the curves were set to 1.

Our previous study demonstrated that an E-box-like sequence (CACGTT) and its downstream region are essential for transcriptional oscillation of *Per2*, a crucial component of molecular clocks [26]. Comparing with other mammalian genome sequences, within the conserved regions among mammalian (human, rhesus, rat, mouse, and opossum) genomes, we found that an E-box-like sequence followed by another non-canonical E-box with 6-base pairs (bp) between the two (Fig. 1A). In addition to *Per2*, we found a similar E-box or E-box-like sequence followed by another E-box-like sequence with an intervening space of 6 bp in *Per1 *and *Per3*, which are other mammalian *period *homologues, in their upstream promoter region close to the transcription start site (TSS). The E-box or E-box-like sequences distal and proximal to TSS were dubbed E1 and E2, respectively, and collectively as the "EE-element" (Fig. 1A). These sequences are well conserved among diverse mammals, such as human, rhesus, rat, and mouse, and even the opossum which is evolutionally located at the farthest branch from humans among the mammals. To examine whether these EE-elements are involved in transcriptional oscillation, we performed the IV-ROMS established in our previous study by using the following constructs: The oligonucleotides of 3 (*hPer1*, *hPer2*, and *hPer3*) EE-elements were inserted upstream of the SV40 promoter in a *luciferase *(*luc*) plasmid, and the constructed plasmids were used to transfect mouse NIH3T3 cells. All 3 EE-elements gave significant oscillation of luciferase activity but the amplitudes were different among the genes (Fig. 1B). The *hPer2 *EE-element displayed the most robust oscillation, whereas the *hPer1 *EE-element gave the lowest amplitude, which correlated with the patterns derived from *luc*-constructs containing the longer promoter regions [26]. The amplitude in circadian oscillation seemed to correlate with the number of mismatches in the E2 sequence. In fact, a mutant *hPer3 *EE-element, in which the GAG at the end of the E2 sequence was replaced with canonical GTG, exhibited a higher amplitude than the wild-type *hPer3 *EE-element (see Additional file 1).

**Figure 2:**
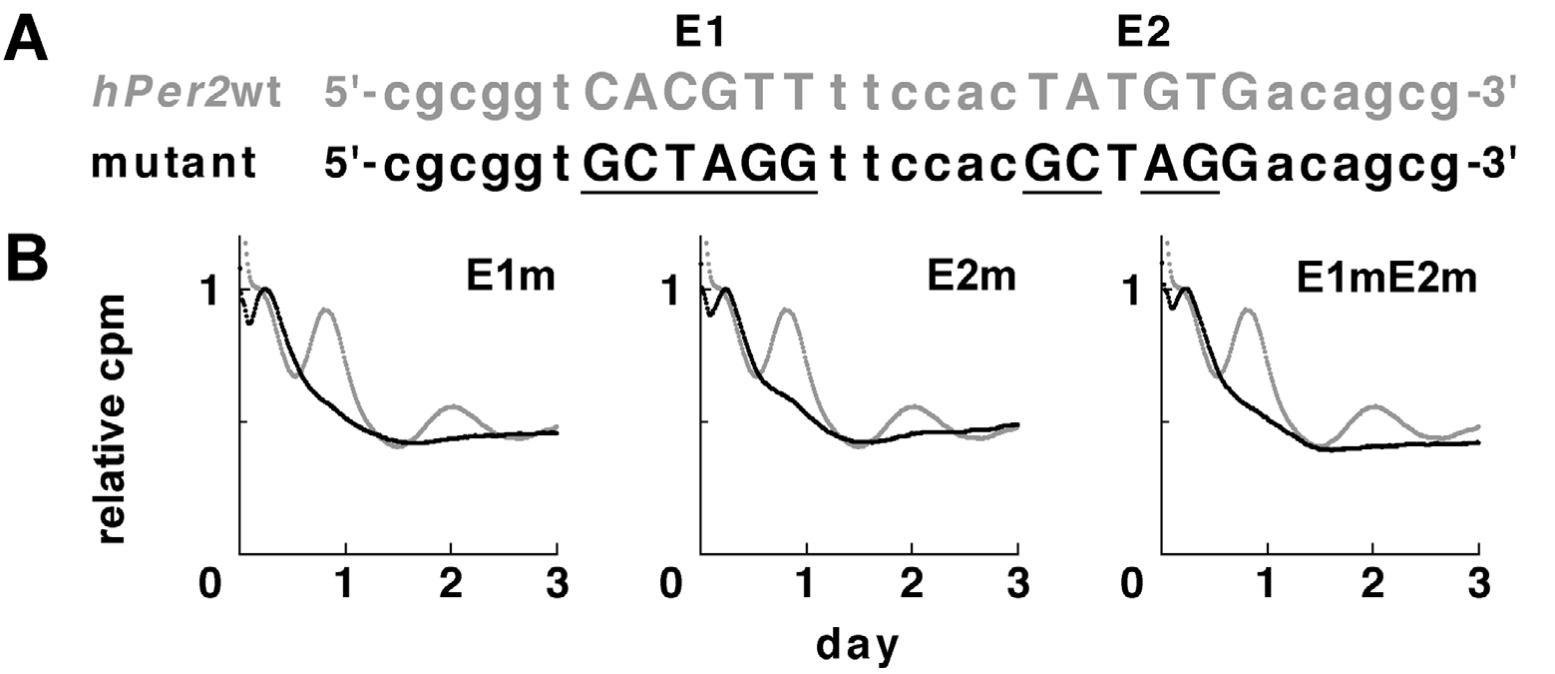
**Effects of EE-element mutants on circadian expression of luciferase**. (A) Wild-type (upper, gray letters) and mutant (bottom, black letters) sequences of *hPer2 *E1 and E2 and their flanking sequences are shown. Underlines indicate sequences changed from the wild-type one. The core sequence within the EE-element is shown in capital letters. (B) EE-element-driven luciferase bioluminescence by IV-ROMS. Gray lines indicate bioluminescence of wild-type *hPer2 *EE-element; and black lines, those of mutants. The abscissa presents "day", and the ordinate indicates "relative luciferase intensity". First peak values of the curves were set to 1.

To investigate whether both E1 and E2 were necessary for generating the cell-autonomous oscillation, we again performed IV-ROMS with the use of the mutant *hPer2 *EE-element constructs. Not only the double E-box-like mutations (E1mE2m) but also the single E-box-like mutation (E1m or E2m) resulted in drastically reduced circadian transcription or led to an almost complete loss of transactivation (Fig. 2). A longer *Per2 *promoter (~2 kb) with double E-element mutants showed that the amplitude was heavily reduced, with circadian transcription hardly being observed (see Additional file 2). These results are similar to those obtained with mutations of 2 ROREs in the *Bmal1 *promoter [21] and suggest that these 2 E-box-like elements function in *Per2 *transactivation.

**Figure 3:**
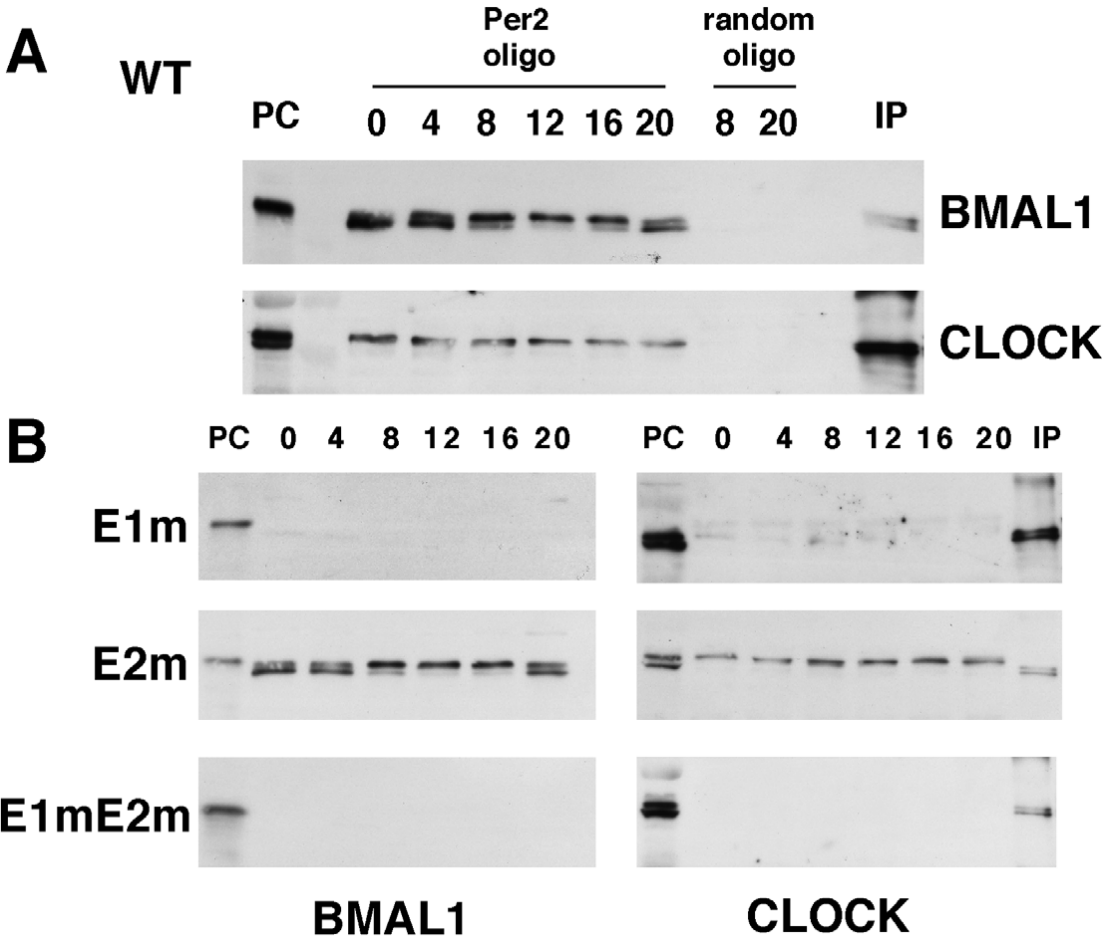
**Pull-down assays by using oligonucleotides**. (A) Both BMAL1 and CLOCK proteins bind to *Per2 *oligonucleotide. BMAL1 is phospholrylated in a circadian manner, whereas no binding to the random oligonucleotide occurs. Mouse liver lysates at CT 16 were electrophoresed and immunoblotted with anti-BMAL1 and anti-CLOCK (PC) and immunoprecipitated by anti-CLOCK and blotted by either BMAL1 or CLOCK (IP). WT; wild-type *hPer2 *oligonucleotide, E1; E1 mutant oligonucleotide, E2; E2 mutant oligonucleotide, E1mE2m; E1, E2 double mutant oligonucleotide. (B) As for BMAL1, the binding to E2m is clearly observed; whereas no binding to either E1m or E1mE2m is detected. For CLOCK, the binding to E2m is observed; however, the binding to E1m is hardly seen and no binding to E1mE2m is evident.

To see whether endogenous clock transcription factors, BMAL1 and CLOCK, could bind to the EE-element, we investigated the binding of endogenous BMAL1 and CLOCK to *hPer2 *double-stranded DNA fragment immobilized on streptoavidin beads (Fig. 3). For this experiment, we raised specific antibodies against BMAL1 (see Additional file 3). Mouse liver lysates were prepared at 4-h intervals and immunoblotted with antibodies against BMAL1 or CLOCK (indicated as PC in Fig. 3A). When the lysates were incubated with the bead-bound *Per2 *oligonucleotides and electrophoresis performed, followed by immunoblotting, bands corresponding to BMAL1 and CLOCK were detected (Fig. 3A). The shifted bands seen correspond to phosphorylated BMAL1; and its phosphorylation level peaked around at CT (circadian time) 12, which is consistent with our previous data obtained with the *Per2 *promoter [26]. In contrast, the oligonucleotides with the same length but having scrambled sequences bound neither BMAL1 nor CLOCK proteins. The amounts of BMAL1 and CLOCK pulled down with mutant E1m or with the double-mutant E1mE2m were drastically reduced or almost undetectable compared with those pulled-down by the wild-type EE-element (Fig. 3B). This result suggests that the endogenous BMAL1 and CLOCK proteins bound to the E1 element but not to E2. On the other hand, when we used the E2m oligonucleotide, precipitated BMAL1 and CLOCK proteins were substantially detected. These results suggest that the binding of CLOCK/BMAL1 to E1 and that of unknown factors to E2 are necessary for cell-autonomous circadian transcription.

**Figure 4:**
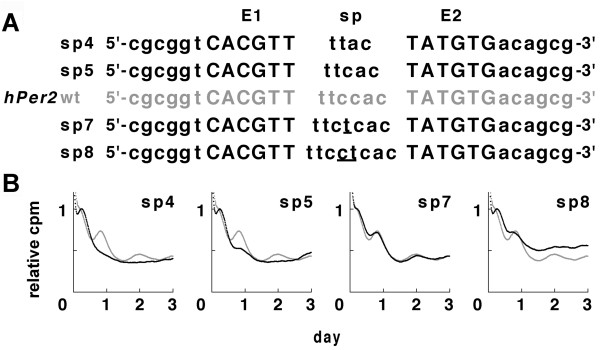
**The space between the 2 E-box-like sequences is critical for cell-autonomous circadian transcription**. (A) Wild-type (sp6; gray letters) and mutant (sp4, 5, 7, 8; solid letters) sequences of the *hPer2 *EE-element and their flanking sequences are shown. Underlines indicate nucleotide(s) inserted into the space of the EE-element. The core sequence within the EE-element is shown in capital letters. (B) EE-element-driven luciferase bioluminescence detected by IV-ROMS. Black lines indicate bioluminescence of mutant *hPer2 *EE-elements; and gray lines, those of the wild type. The abscissa presents "day"; the ordinate shows "relative luciferase intensity". First peak values of the curves were set to 1.

We next examined the length of the space between E1 and E2, because the spaces between two E-box-like elements were 6 bp in all of 3 *Per*s EE-elements (Fig. 1A). To see whether the space length was crucial for cell-autonomous circadian transcription, we constructed different spaces (4~8 bp) between E1 and E2 of the *hPer2 *EE-element and performed IV-ROMS (Fig. 4). sp7, in which there were 7 bp spacing nucleotides between the 2 E-elements, yielded a circadian rhythmicity of *Per2 *transcription but with a slightly diminished amplitude compared with the wild-type oligonucleotide. The amplitude of sp5 and sp8 (5-bp and 8-bp space, respectively) or sp4 (4-bp space) displayed almost no or no oscillation of *Per2*, respectively. These results demonstrate that the spacing between the 2 E-elements was critical for rhythm generation and that the 6-bp (or 7-bp) space was necessary for cell-autonomous transcriptional oscillation. Thus the EE-element for circadian rhythm may be defined as follows: 1) E1 and E2 allow 1 and 2 base mismatches, respectively, and 2) the space between the 2 E-elements is 6 or 7 bp (Table 1).

**Table 1:** The criteria for the EE-element. Criteria for the EE-element are as follow: 1) 2 E-boxes, close to each other, are needed, 2) E1 E-box allows 1 base mismatch, E2 E-box allows 2 base mismatches, 3) the spacing between 2 E-boxes is 6 or 7 bp, and 4) the element is located within 1 kb upstream region from the TSS.

**E-box**	**space**	**E-box**
C A C G T G	6 or 7 bp	C A C G T G
0~1 mismatch		0~2 mismatch

Previous studies [28-30] in which clock or clock-controlled genes were extensively examined at the transcriptional level by DNA microarrays revealed that a total of 1551 genes exhibited circadian oscillation. So we examined how many genes contained the EE-element that met our criteria described above in their promoter regions within 1 kb upstream from the TSS. As a consequence of *in silico *analysis, 30 EE-elements in 29 genes were found among 1510 genes, which sequences are available from the VISTA database (Table 2). The genes that appeared in different papers and organs were clock and clock-controlled genes, such as *Nr1d1 *(also known as *Rev-erbα*), *Dbp*, *Avp *and *Tef*, besides *Per2*. This result strongly suggests that not a single E-box but a direct repeat of E-box-like elements is essential for the generation of cell-autonomous circadian oscillation. To confirm this, by using IV-ROMS we investigated whether the EE-element in *Dbp *was also required for its rhythmicity. The *hDbp *EE-element showed circadian oscillation as well as the *hPer2 *one (see Additional file 4). The mutants of E1 and/or E2 in the EE-element as well as the space mutants, sp4, sp5, and sp8 displayed no rhythmicity (see Additional file 4), which is consistent with the results of *Per2 *described above.

**Table 2:** The EE-elements in clock and clock-controlled genes. Clock and clock-controlled genes containing putative EE-elements (A; [28], B; [29], C; [30]). "Phase" means peak time. Phase 2, the peak value = CT 0.1~4.0; phase 6, CT 4.1~8.0; phase 10, CT 8.1~12; phase 14, CT 12.1~16; phase 18, CT 16.1~20; phase 22, CT 20.1~24. "pos" indicates the position of the EE-element from the TSS. Underlines indicate nucleotides matched to the canonical E-box.

**Paper**	**Organ**	**Phase**	**Gene**	**pos**	**mouse**		**human**	
					
					**sequence**	**sp**	**sequence**	**sp**
A	SCN	10	Per2	-23	CACGTTTTCCACTATGTG	6	CACGTTTTCCACTATGTG	6
A	Liver	14		-23				
B	Liver	14		-23				
B	Heart	10		-23				
C	SCN	10		-23				
C	Liver	14		-23				
A	SCN	18	Nr1d1	-878	CACGTGAAGCTCTCACGTT	7	CACGTGAAGCCCTCACGTT	7
A	Liver	6		-878				
B	Liver	6		-878				
B	Heart	6		-878				
C	Liver	6		-878				
B	Liver	6	Dbp	-606	CACGCGCAAAGCCATGTG	6	CACGAGCAGAGCCATGTG	6
B	Heart	10		-606				
C	SCN	6		-606				
B	Liver	10	Tef	-529	CACGTGCAGAGCCTCGTG	6	CACGTGCGGCGCCTCGTG	6
B	Liver	10		-208	CACCTGGCCCAGCACGTG	6	CACCTGGCCCCGCACGTG	6
B	Heart	10		-529				
B	Heart	10		-208				
A	Liver	10	Foxo3a	-97	CACGCGCACTCCCACACG	6	CACGCGCACTCACACACG	6
B	Liver	10		-97				
C	Liver	10		-97				
A	Liver	10	3732413I11Rik	-307	CACGTGGTCCCAGCAGGTG	7	CACGTGGTCCCTGCAGGTG	7
C	Liver	10		-307				
A	Liver	18	Tfdp1	-916	CACGTCACCGCGCCGCGCG		CACGTCACCGCGCCGCGCG	7
B	Liver	14		-916				
B	Liver	18	Tfrc	-127	TACGTGCGGAAGGAAGTG	6	TACGTGCCTCAGGAAGTG	6
B	Heart	2		-127				
B	Liver	22	Sox3	-200	CAGGTGAGAGAAGCCCGCG	7	CAGGTGCGAGAAGCCCGCG	7
B	Heart	18		-200				
B	Heart	2	MGI:1351465	-387	CACGGGCCCCACCTTGTG	6	CACGGGCCCCACCTTGTG	6
C	SCN	6	Avp	-186	CACGTGTGTCCCCAGATG	6	CACGTGTGTCCCCAGATG	6
A	SCN	14	Serpine1	-179	CACGTGTCCCAGCAAGTC	6	CACATGCCTCAGCAAGTC	6
B	Liver	6	Wwp2	-33	CACGTGACCCGGCCCGAG	6	CACGTGACCCGGCCCGAG	6
C	SCN	18	Hnrpm	-42	CACGTGAGCGCGCAGGCG	6	CACGTGGGCGCGCAGGCG	6
B	Heart	6	Snx2	-41	CACGTGACAGGTCCGCGAG	7	CACGTGACGGGTCCGCGAG	7
A	SCN	18	Spag7	-238	CACGAGCACTTTCTACTTG	7	CACGGGCACTTTCTACTTG	7
A	SCN	10	Pabpn1	-438	CACCTGTCGCACAACGGG	6	CACCTGTCACGAAACGAG	6
B	Heart	18	Zic2	-415	CAGGTGGAGCGGCGGGTG	6	CAGGTGGAGCCGCTGGTG	6
A	SCN	18	Cacna2d3	-37	CAAGTGAGCCGGGCGCGCG	7	CAAGTGAGCCGGGCGCGCG	7
A	SCN	10	Cog8	-705	CACGTGCAAGCGGAACTTG	7	GACGTGCAACCGAAACTTG	7
B	Heart	2	Col4a2	-641	CACCTGGCCGTGCCACCCG	7	CACCTGGCCGTGCCACGCG	7
C	SCN	14	Ube2s	-117	AACGTGCGCGTTGACGTA	6	AACGTGCGCGCTGACGTC	6
A	SCN	10	Igsf4a	-584	GACGTGCAAAGCACGCATG	7	GACGTGCAAAGCACGCATG	7
C	SCN	18	Plekha1	-31	CGCGTGCGCAGTGCGCGGG	7	CGCGTGCGCAGTGCGCGGG	7
B	Heart	2	Qk	-352	CCCGTGGGTGCGCACGCG	6	CCCGTGGGTGCGCACGCG	6
C	SCN	6	Rexo2	-27	CCCGTGGGTTTGCGACGTT	7	CCCGTGGGTTTGCGACGTT	7
B	Liver	10	Trfp	-683	CGCGTGGCGCGCTCTCGCG	7	CGCGTGGTGCGTTCCCGCG	7
C	Liver	18	BC014685	-186	CCCGTGACATTGCCGCGGG	7	CCCGTGACGCGGGCGCGGG	7
B	Heart	2	Ptma	-23	CGCGTGAGTCCCCCACTGG	7	CGCGTGAGTCCCCCACTGG	7

## Discussion

Among the genes identified by *in silico *analysis (Table 2), arginine vasopressin (*Avp*) is a neuropeptide physiologically synthesized in the hypothalamus in a circadian manner but pathologically expressed by small-cell lung cancer. In the latter case, upstream stimulatory factor (USF), another bHLH transcription factor, activates the vasopressin promoter via the adjacent non-canonical E-boxes in tandem with a 6-nucleotides space between them and the second E-box is important for full enhancer function of the first E-box [31]. Furthermore, hypoxia-inducible factor 1 (HIF-1), a bHLH-PAS transcription factor, has recently been reported to regulate some hypoxia-inducible genes such as vascular endothelial growth factor (VEGF) through an HIF-1 binding site and its downstream HIF-1 ancillary sequence, both of which are non-canonical E-boxes [32]. The space between these motifs in this case is 8 nucleotides and is crucial for activity of promoter. Not only mammalian genes but also fly clock genes contain a direct repeat of E-box-like elements, as discovered in this study. The *Drosophila timeless *promoter includes an E-box-like sequence dubbed TER1 and is considered as an EE-element together with its 7-bp downstream canonical E-box. This TER1 plays a major role in *tim *transcription [33]. These reports in addition to this study demonstrate that not a single E-box but an EE-element is crucial for transcriptional activation by certain bHLH transcription factors.

**Figure 5:**
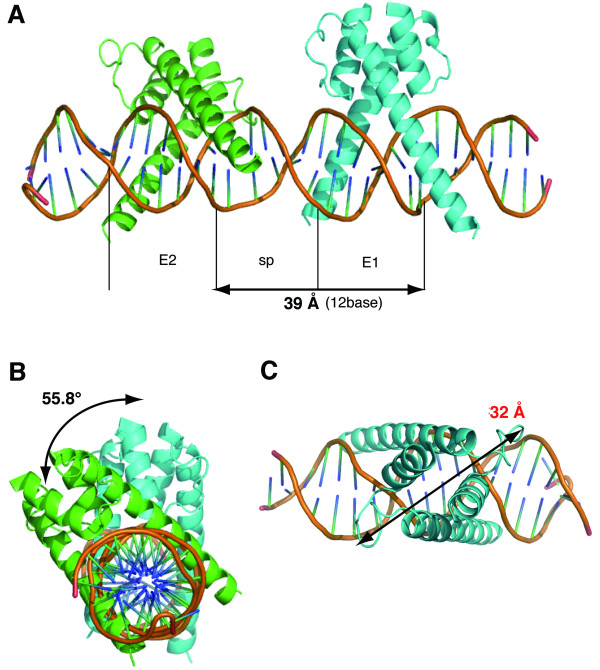
**Structural model for the binding of bHLH transcription factors to DNA**. (A) Since 1 turn of double helix DNA is approximately 34 Å and requires 10.4 nucleotides, 1 EE-element, which consists of 12 nucleotides, spans 39.2 Å (34/10.4 × 12). (B) Two binding faces form a 55.8-degree angle (360/10.4 × 12–360). (C) The maximum dimension of the bHLH structure, which lies along the DNA, is about 32 Å, which is within the 39 Å calculated above and allows the binding. Green indicates a hetero complex of transcription factors such as BMAL1 and CLOCK; and light blue, unknown factors.

Our results suggest that 2 dimeric complexes of transcription factors bind to the 2 E-box-like elements with a short distance between them, such as 6 nucleotides. To support this possibility, we simulated this binding of bHLH transcription factors to the EE-element on the DNA by using the crystal structure of the bHLH domain of the Myc-Max heterodimer bound to DNA (PDB ID: 1NKP) [34]. Since 1 turn of double-helix DNA is approximately 34 Å and requires 10.4 nucleotides, the distance from the beginning of E1 to the end of space, which consists of 12 nucleotides, spans 39.2 Å (34/10.4 × 12, Fig. 5A). Two binding faces form a 55.8-degree angle (360/10.4 × 12–360, Fig. 5B). The maximum dimension of the bHLH structure, which lies along the DNA, is about 32 Å (Fig. 5C). This dimension is within the 39 Å calculated above and allows the binding. When the space is 5 nucleotides, for example, the distance between 2 complexes shortens approximately 3 Å horizontally compared with the case of 6 nucleotides; however, the angle between them becomes 20.6°. This precludes the possibility that the bHLH structure binds to the EE-element, because 2 whole structures including PAS domains may collide with each other. This simulation supports our contention that 6 nucleotides between 2 E-box-like elements, not 5 nucleotides, allow the binding of bHLH transcription factors to the EE-element on the DNA.

As a candidate for unknown factors that bind to E2, another type of transcription factor can be a possible candidate as well as the bHLH family transcription factor including HIF1 [35] or the bZip family transcription factor E4BP4 (also known as NFIL3) [36]. Our preliminary EMSA suggests that some proteins may bind to E2 (see Additional file 5). Alternatively, different forms of the CLOCK and BMAL1 complex can be involved in this circadian transcription. The circadian transcriptional complex includes at least CLOCK, NPAS2, BMAL1, PERs and CRYs, probably in different combinations, and these proteins can be posttranslationally modified [37]. While E1 element is sufficient for binding of CLOCK and BMAL1, it is possible that binding to E2 can occur only after CLOCK and/or BMAL1 originally binds to E1. Thus E2 can be necessary for the binding of different forms of the complex or perhaps the binding to E2 is important for complex activation. Further studies, including identification of unknown factors or the circadian transcription complex and their crystal structures, will be required to elucidate the precise mechanism of transcriptional activation via an EE-element.

## Conclusion

Here we identified a direct repeat of E-box-like elements consisting of a canonical E-box or non-canonical E-box in tandem with an E-box-like element, having a short interval of 6 base pairs between them. Although other regions outside the EE-element seem to be active in robust circadian oscillation with high amplitude [38], the EE-element identified in this study, not a single E-box, is the minimal required element for the generation of cell-autonomous transcriptional oscillation of clock and clock-controlled genes.

## Methods

### Cell culture

NIT3T3 cells were grown at 37°C and 5% CO_2 _in Dulbecco's modified Eagle's medium (1.0 g/L glucose) with L-Gln and sodium pyruvate (DMEM, Nacalai tesque, Kyoto, Japan) supplemented with 10% fetal bovine serum (FBS; ICN Biomedicals, Inc.) and antibiotics.

### Antibody

Rabbit BMAL1 polyclonal antibody was raised against glutathione S-transferase (GST) fused to the amino terminus of BMAL1 (amino acids 1–99). The antiserum was purified through GST and GST-BMAL1 columns. Also, BMAL1 and E4BP4 antibodies were kindly donated by Drs. Teruya Tamaru [39] and Toshiya Inaba [40], respectively. CLOCK antibody (S-19; sc-6927) was purchased from Santa Cruz Biotechnology, Inc.

### Construction of the EE-element::SV40 promoter-luciferase plasmid

The oligonucleotide sequences used in this study are indicated in Additional file 6. Each fragment of the double-stranded oligonucleotides of the *hPer *genes was cloned into a modified pGL3 (R2.2) promoter vector digested with *Sac*I-*Nhe*I. To construct the modified pGL3 (R2.2) promoter vector plasmid, we excised the SV40 promoter fragment from the pGL3 promoter vector (Promega) by digestion with *Bgl*II-*Nco*I and ligated it into the compatible site of pGL3 (R2.2) basic vector (Promega). To rule out the possibility that 2 E-box-like elements in multiple cloning sites of this vector possessed a binding potential for CLOCK/BMAL1, these sequences were removed.

### IV-ROMS (*in vitro *real-time oscillation monitoring system)

NIH3T3 cells at 1.0 × 10^5 ^plated in Opti-MEM (GIBCO) supplemented with 10% FBS in 35-mm dishes were transfected with the desired plasmids by using LipofectAMINE and plus reagent (Invitrogen). Twenty-four hours after transfection, the medium was exchanged for serum-rich medium (DMEM supplemented with 50% newborn calf serum [NBS; JRH]), and 2 h later this medium was replaced with Opti-MEM supplemented with 1% FBS and 0.1 mM luciferin/10 mM HEPES (pH 7.2). Bioluminescence was measured by using IV-ROMS as described previously [25].

### Oligonucleotide pull-down experiment

The oligonucleotide pull-down experiment was performed as described previously [21,26]. Mouse liver lysate was prepared at 4-h intervals by homogenizing liver in ice-cold PBS with 2 mM EDTA and 10 μg/ml aprotinin/leupeptin/pepstatin, and 1 mM PMSF as proteinase inhibitors (PIs). The pellet was sequentially incubated in hypotonic buffer (50 mM Tris-HCl pH 7.4, 2 mM MgCl_2_, 1 mM EDTA, 1 mM DTT, and PIs) and incubation buffer (50 mM Tris-HCl pH 7.4, 50 mM NaCl, 2 mM EDTA, 10% glycerol, 1% NP40, 1 mM DTT, 100 mM NaF, 2 mM Na_3_VO_4_, 10 mM Na_4_P_2_O_7_, and PIs). After centrifugation, the supernatant was transferred to a new tube and the remaining pellet was sonicated; and following centrifugation its supernatant combined with the original supernatant as the nuclear extract. The nuclear extracts were incubated with a biotinylated oligonucleotide (see Additional file 6) that had been immobilized on streptavidin-Sepharose beads (Amersham) for 1 h at 4°C. After having been washed with incubation buffer, the resultant complex was subjected to immunoblot analysis. The surgical and experimental procedures performed on mice were approved by the OBI Animal Research Committee.

### *In silico *analysis of the EE-element

The gene list for this analysis was derived from the reports of microarray analyses [28-30]. The genes were classified by the phase of the expression peak and organ in which each gene was expressed (phase 2, the peak time = CT 0.1~4.0; phase 6, CT 4.1~8.0; phase 10, CT 8.1~12; phase 14, CT 12.1~16; phase 18, CT 16.1~20; phase 22, CT 20.1~24). The aligned sequences spanning from 1 kb upstream of the transcription start site (TSS) to the 3' untranslated region in each of human and mouse genes were obtained from the VISTA genome browser (VISTA Browser, VGB2, [41], version: Human, May 2004; Mouse, Aug. 2006), which has pre-computed alignments of whole genome assemblies. Fuzznuc (EMBOSS, [42]), a tool for nucleic acid pattern searching, was used for the first screening; each aligned sequence from VISTA was searched with the following sequence: E-box (CACGTG, 0~1 base mismatch) + 6 or 7 arbitrary bases + E-box (CACGTG, 0~2 base mismatches). The conserved EE-elements between human and mouse were listed. Subsequently, the EE-elements within 1 kb upstream region from TSS were listed in Table 2.

## Authors' contributions

YN and MY carried out experiments and drafted the manuscript. AT discussed the results. MY, HS, and TY carried out the in silico study. AY participated in the coordination and provided financial support. TN carried out the structural design. TT participated in the experimental design and coordination and wrote the paper. All authors read and approved the final manuscript.

## Supplementary Material

Additional file 1 **Effect of the E2 mutant in *hPer3 *on circadian expression of luciferase**. Wild-type (gray letters) and mutant (black letters) sequences of *hPer3 *EE-element and their flanking sequences are shown in upper panel. The core sequence within the EE-element is shown in capital letters. Bioluminescence monitoring of the *hPer3 *EE-element-*Luc *construct was performed by using IV-ROMS (black; mutant, gray; wild-type). The abscissa presents "day"; and the ordinate shows "relative luciferase intensity". First peak values of the curves were set to 1. Click here for file

Additional file 2 **Effect of the EE-element mutant in *Per2 *promoter on circadian expression of luciferase**. Bioluminescence monitoring of the *Per2 *promoter-*Luc *construct was performed by IV-ROMS (black; mutant, gray; wild-type). The abscissa presents "day"; and the ordinate indicates "relative luciferase intensity". First peak values of the curves were set to 1. Click here for file

Additional file 3 **Characterization of BMAL1 antibody**. (A) Western blotting. Two bands at around 70–80 kDa that correspond to BMAL1 were observed in mouse liver lysates at 4-hr intervals. CT, circadian time; arrows, BMAL1. (B) Immunoprecipitated and blotted with BMAL1 antibody. WT, liver lysate from wild-type mouse; KO, liver lysate from BMAL1-deficeient mouse. CIP, calf intestine alkaline phosphatase; arrows, BMAL1; *, a phosphorylated band. Click here for file

Additional file 4 **The EE-element of *hDbp *promoter is sufficient for circadian rhythm generation**. (A) Wild-type and mutant sequence of *hDbp *E1 and E2 and their flanking region are shown. The core sequence within the EE-element is shown in capital letters. (B) EE-element-driven luciferase bioluminescence by IV-ROMS. Gray lines indicate bioluminescence of wild type *hDbp *EE-element; and black lines show those of mutants. The abscissa presents "day"; and the ordinate shows "relative luciferase intensity". First peak values of the curves were set to 1. (C) Wild-type (sp6; gray letters) and mutant (sp4, 5, 7, 8; solid letters) sequences of *hDBP *EE-element and their flanking sequences are shown. Underlines indicate inserted nucleotides into space of the EE-element. The core sequence within the EE-element is shown in capital letters. (D) EE-element-driven luciferase bioluminescence by IV-ROMS. Black lines indicate bioluminescence of the mutant *hDbp *EE-elements; and the gray lines show those of wild type. The abscissa presents "day"; and the ordinate shows "relative luciferase intensity". First peak values of the curves were set to 1. Click here for file

Additional file 5 **Some protein may bind to E2**. Electrophoretic mobility shift assay (EMSA) shows that some protein from cellular lysates binds to E2 (left). The bands from liver lysates in E1m were attenuated with excess of E1m, but not in E2m (right). Click here for file

Additional file 6 **The information of constructs and genes used in this study**. Upper panel shows the construct sequences used in this study. Middle panel demonstrates the oligonucleotides used in Figure 2C. Bottom panel shows the accession numbers of *Per*s and *Dbp *used. Click here for file

## References

[B1] Schibler U, Sassone-Corsi P (2002). A web of circadian pacemakers. Cell.

[B2] Hastings MH, Reddy AB, Maywood ES (2003). A clockwork web: circadian timing in brain and periphery, in health and disease. Nat Rev Neurosci.

[B3] Yamamoto T, Nakahata Y, Soma H, Akashi M, Mamine T, Takumi T (2004). Transcriptional oscillation of canonical clock genes in mouse peripheral tissues. BMC Mol Biol.

[B4] Schibler U (2005). The daily rhythms of genes, cells and organs. Biological clocks and circadian timing in cells. EMBO Rep.

[B5] Fu L, Lee CC (2003). The circadian clock: pacemaker and tumour suppressor. Nat Rev Cancer.

[B6] Bell-Pedersen D, Cassone VM, Earnest DJ, Golden SS, Hardin PE, Thomas TL, Zoran MJ (2005). Circadian rhythms from multiple oscillators: lessons from diverse organisms. Nat Rev Genet.

[B7] Ko CH, Takahashi JS (2006). Molecular components of the mammalian circadian clock. Hum Mol Genet.

[B8] Wijnen H, Young MW (2006). Interplay of circadian clocks and metabolic rhythms. Annu Rev Genet.

[B9] Ueda HR, Hayashi S, Chen W, Sano M, Machida M, Shigeyoshi Y, Iino M, Hashimoto S (2005). System-level identification of transcriptional circuits underlying mammalian circadian clocks. Nat Genet.

[B10] Gekakis N, Staknis D, Nguyen HB, Davis FC, Wilsbacher LD, King DP, Takahashi JS, Weitz CJ (1998). Role of the CLOCK protein in the mammalian circadian mechanism. Science.

[B11] Kume K, Zylka MJ, Sriram S, Shearman LP, Weaver DR, Jin X, Maywood ES, Hastings MH, Reppert SM (1999). mCRY1 and mCRY2 are essential components of the negative limb of the circadian clock feedback loop. Cell.

[B12] Hida A, Koike N, Hirose M, Hattori M, Sakaki Y, Tei H (2000). The human and mouse Period1 genes: five well-conserved E-boxes additively contribute to the enhancement of mPer1 transcription. Genomics.

[B13] Jin X, Shearman LP, Weaver DR, Zylka MJ, de Vries GJ, Reppert SM (1999). A molecular mechanism regulating rhythmic output from the suprachiasmatic circadian clock. Cell.

[B14] Ripperger JA, Shearman LP, Reppert SM, Schibler U (2000). CLOCK, an essential pacemaker component, controls expression of the circadian transcription factor DBP. Genes Dev.

[B15] Etchegaray JP, Lee C, Wade PA, Reppert SM (2003). Rhythmic histone acetylation underlies transcription in the mammalian circadian clock. Nature.

[B16] Triqueneaux G, Thenot S, Kakizawa T, Antoch MP, Safi R, Takahashi JS, Delaunay F, Laudet V (2004). The orphan receptor Rev-erbalpha gene is a target of the circadian clock pacemaker. J Mol Endocrinol.

[B17] Ripperger JA, Schibler U (2006). Rhythmic CLOCK-BMAL1 binding to multiple E-box motifs drives circadian Dbp transcription and chromatin transitions. Nat Genet.

[B18] Preitner N, Damiola F, Lopez-Molina L, Zakany J, Duboule D, Albrecht U, Schibler U (2002). The orphan nuclear receptor REV-ERBalpha controls circadian transcription within the positive limb of the mammalian circadian oscillator. Cell.

[B19] Sato TK, Panda S, Miraglia LJ, Reyes TM, Rudic RD, McNamara P, Naik KA, FitzGerald GA, Kay SA, Hogenesch JB (2004). A functional genomics strategy reveals Rora as a component of the mammalian circadian clock. Neuron.

[B20] Nakajima Y, Ikeda M, Kimura T, Honma S, Ohmiya Y, Honma K (2004). Bidirectional role of orphan nuclear receptor RORalpha in clock gene transcriptions demonstrated by a novel reporter assay system. FEBS Lett.

[B21] Akashi M, Takumi T (2005). The orphan nuclear receptor RORalpha regulates circadian transcription of the mammalian core-clock Bmal1. Nat Struct Mol Biol.

[B22] Albrecht U, Sun ZS, Eichele G, Lee CC (1997). A differential response of two putative mammalian circadian regulators, mper1 and mper2, to light. Cell.

[B23] Takumi T, Matsubara C, Shigeyoshi Y, Taguchi K, Yagita K, Maebayashi Y, Sakakida Y, Okumura K, Takashima N, Okamura H (1998). A new mammalian period gene predominantly expressed in the suprachiasmatic nucleus. Genes Cells.

[B24] Zheng B, Larkin DW, Albrecht U, Sun ZS, Sage M, Eichele G, Lee CC, Bradley A (1999). The mPer2 gene encodes a functional component of the mammalian circadian clock. Nature.

[B25] Nakahata Y, Akashi M, Trcka D, Yasuda A, Takumi T (2006). The in vitro real-time oscillation monitoring system identifies potential entrainment factors for circadian clocks. BMC Mol Biol.

[B26] Akashi M, Ichise T, Mamine T, Takumi T (2006). Molecular mechanism of cell-autonomous circadian gene expression of Period2, a crucial regulator of the mammalian circadian clock. Mol Biol Cell.

[B27] Yoo SH, Ko CH, Lowrey PL, Buhr ED, Song EJ, Chang S, Yoo OJ, Yamazaki S, Lee C, Takahashi JS (2005). A noncanonical E-box enhancer drives mouse Period2 circadian oscillations in vivo. Proc Natl Acad Sci U S A.

[B28] Panda S, Antoch MP, Miller BH, Su AI, Schook AB, Straume M, Schultz PG, Kay SA, Takahashi JS, Hogenesch JB (2002). Coordinated transcription of key pathways in the mouse by the circadian clock. Cell.

[B29] Storch KF, Lipan O, Leykin I, Viswanathan N, Davis FC, Wong WH, Weitz CJ (2002). Extensive and divergent circadian gene expression in liver and heart. Nature.

[B30] Ueda HR, Chen W, Adachi A, Wakamatsu H, Hayashi S, Takasugi T, Nagano M, Nakahama K, Suzuki Y, Sugano S, Iino M, Shigeyoshi Y, Hashimoto S (2002). A transcription factor response element for gene expression during circadian night. Nature.

[B31] Coulson JM, Fiskerstrand CE, Woll PJ, Quinn JP (1999). E-box motifs within the human vasopressin gene promoter contribute to a major enhancer in small-cell lung cancer. Biochem J.

[B32] Kimura H, Weisz A, Ogura T, Hitomi Y, Kurashima Y, Hashimoto K, D'Acquisto F, Makuuchi M, Esumi H (2001). Identification of hypoxia-inducible factor 1 ancillary sequence and its function in vascular endothelial growth factor gene induction by hypoxia and nitric oxide. J Biol Chem.

[B33] McDonald MJ, Rosbash M, Emery P (2001). Wild-type circadian rhythmicity is dependent on closely spaced E boxes in the Drosophila timeless promoter. Mol Cell Biol.

[B34] Nair SK, Burley SK (2003). X-ray structures of Myc-Max and Mad-Max recognizing DNA. Molecular bases of regulation by proto-oncogenic transcription factors. Cell.

[B35] Hogenesch JB, Gu YZ, Jain S, Bradfield CA (1998). The basic-helix-loop-helix-PAS orphan MOP3 forms transcriptionally active complexes with circadian and hypoxia factors. Proc Natl Acad Sci U S A.

[B36] Ohno T, Onishi Y, Ishida N (2007). A novel E4BP4 element drives circadian expression of mPeriod2. Nucleic Acids Res.

[B37] Lee C, Etchegaray JP, Cagampang FR, Loudon AS, Reppert SM (2001). Posttranslational mechanisms regulate the mammalian circadian clock. Cell.

[B38] Munoz E, Brewer M, Baler R (2002). Circadian Transcription. Thinking outside the E-Box. J Biol Chem.

[B39] Tamaru T, Isojima Y, van der Horst GT, Takei K, Nagai K, Takamatsu K (2003). Nucleocytoplasmic shuttling and phosphorylation of BMAL1 are regulated by circadian clock in cultured fibroblasts. Genes Cells.

[B40] Kuribara R, Kinoshita T, Miyajima A, Shinjyo T, Yoshihara T, Inukai T, Ozawa K, Look AT, Inaba T (1999). Two distinct interleukin-3-mediated signal pathways, Ras-NFIL3 (E4BP4) and Bcl-xL, regulate the survival of murine pro-B lymphocytes. Mol Cell Biol.

[B41] VGB2 http://pipeline.lbl.gov/cgi-bin/gateway2.

[B42] EMBOSS http://bioweb.pasteur.fr/docs/EMBOSS/fuzznuc.html.

