# Supplementary figures and images for: A direct repeat of E-box-like elements is required for cell-autonomous circadian rhythm of clock genes
Source: BMC Mol Biol. 2008 Jan 4;9:1. doi: 10.1186/1471-2199-9-1 (PMC2254435; doi:10.1186/1471-2199-9-1)

E1

E2

*hPer3*wt 5'-gaccggCACGCGgcgagcCTCGAGactgcg-3'

GTGmut 5'-gaccggCACGCGgcgagcCTCGTGactgcg-3'

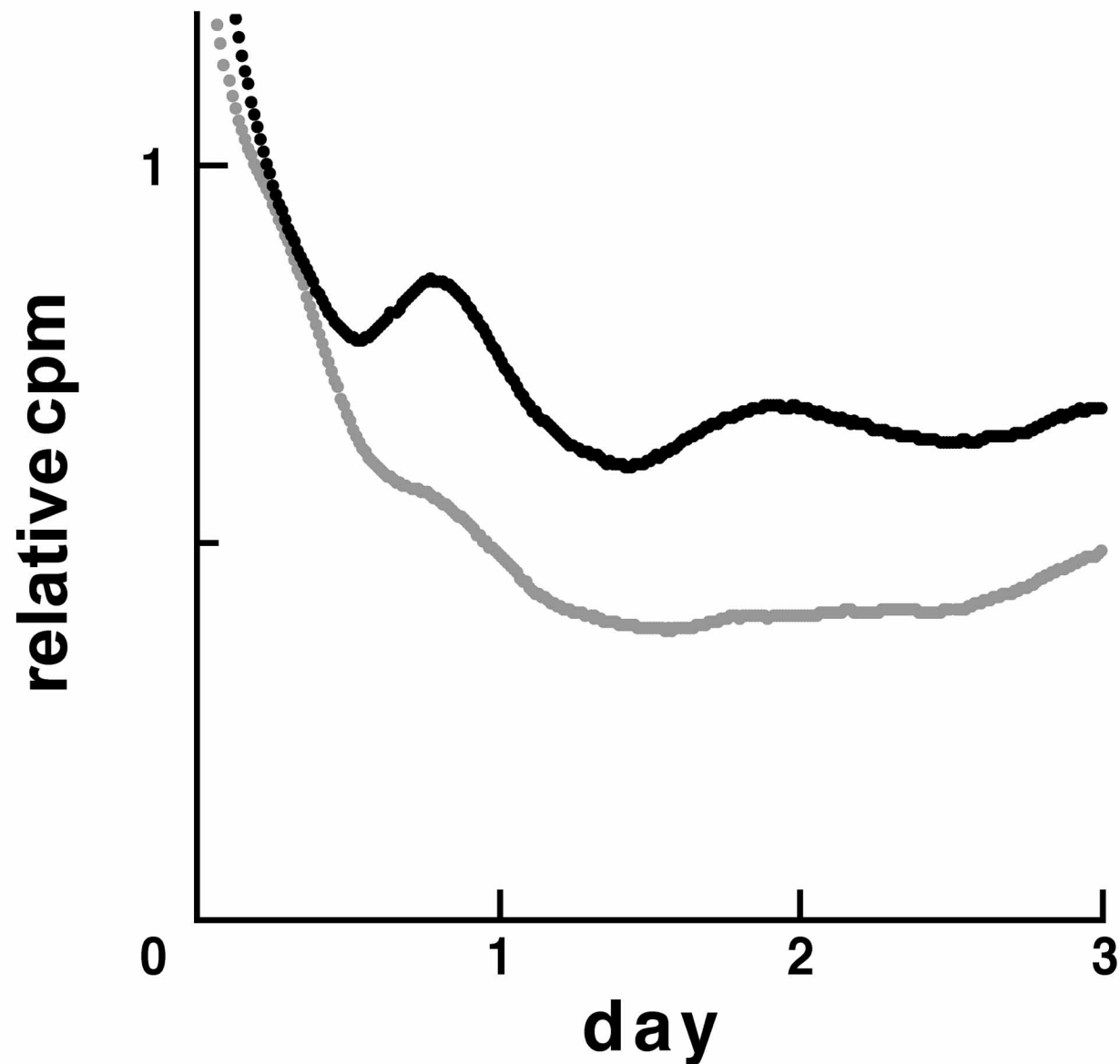

Supplement: Additional file 1 — Effect of the E2 mutant in hPer3 on circadian expression of luciferase. Wild-type (gray letters) and mutant (black letters) sequences of hPer3 EE-element and their flanking sequences are shown in upper panel. The core sequence within the EE-element is shown in capital letters. Bioluminescence monitoring of the hPer3 EE-element-Luc construct was performed by using IV-ROMS (black; mutant, gray; wild-type). The abscissa presents "day"; and the ordinate shows "relative luciferase intensity". First peak values of the curves were set to 1. [file 1471-2199-9-1-S1.pdf]

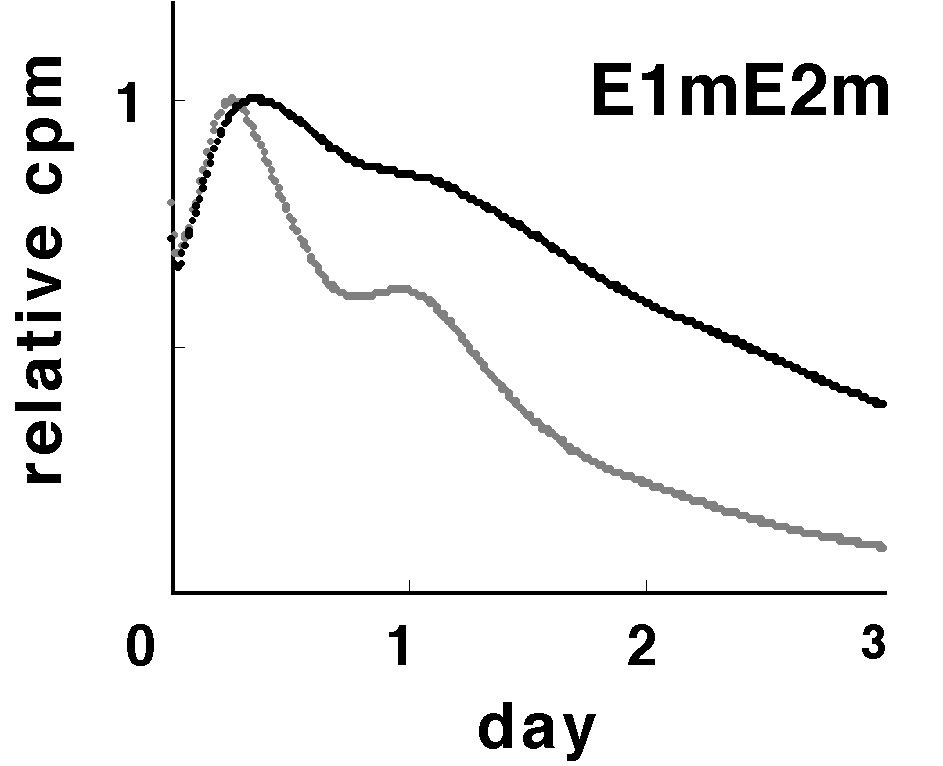

Supplement: Additional file 2 — Effect of the EE-element mutant in Per2 promoter on circadian expression of luciferase. Bioluminescence monitoring of the Per2 promoter-Luc construct was performed by IV-ROMS (black; mutant, gray; wild-type). The abscissa presents "day"; and the ordinate indicates "relative luciferase intensity". First peak values of the curves were set to 1. [file 1471-2199-9-1-S2.tiff]

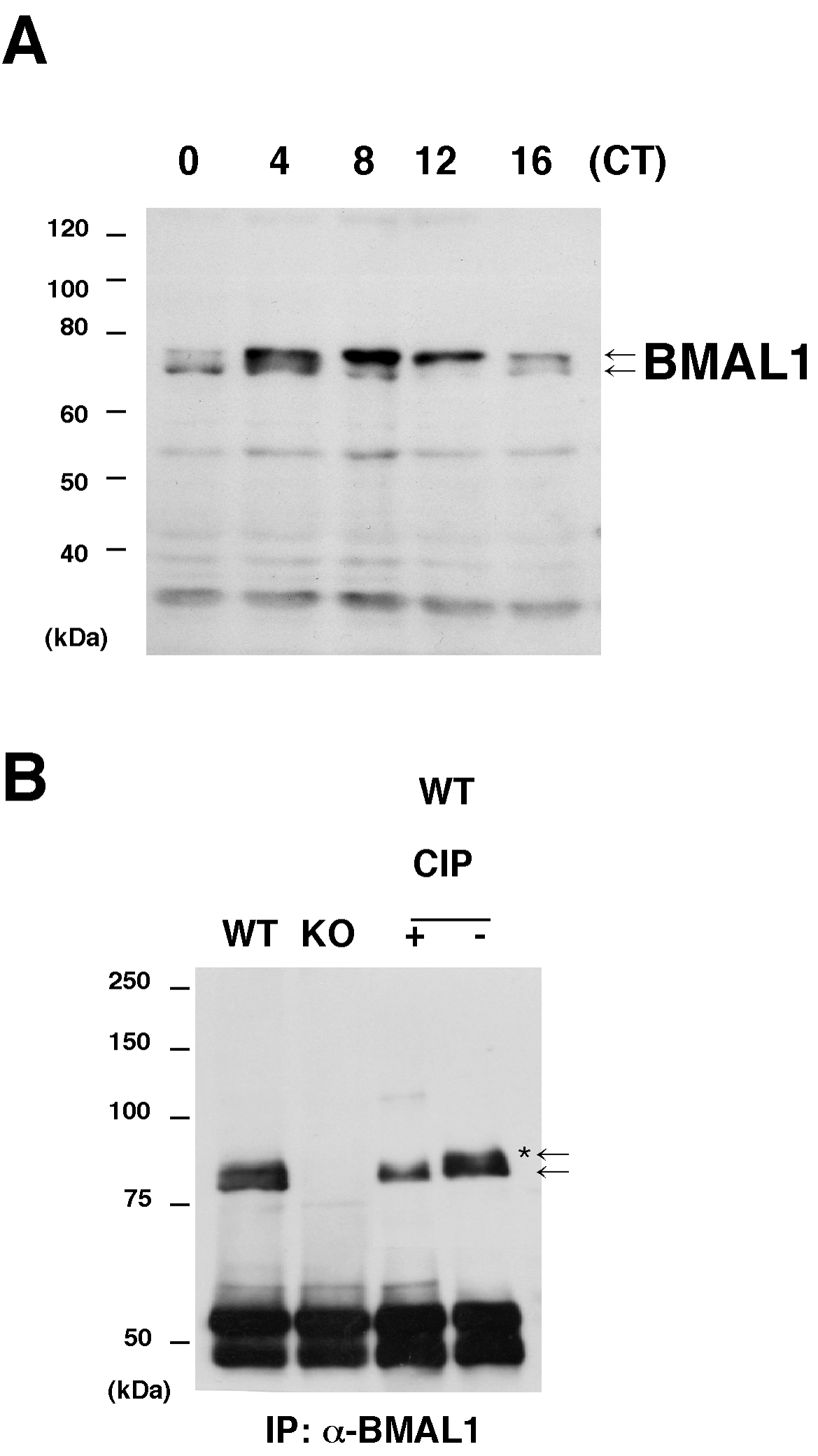

Supplement: Additional file 3 — Characterization of BMAL1 antibody. (A) Western blotting. Two bands at around 70–80 kDa that correspond to BMAL1 were observed in mouse liver lysates at 4-hr intervals. CT, circadian time; arrows, BMAL1. (B) Immunoprecipitated and blotted with BMAL1 antibody. WT, liver lysate from wild-type mouse; KO, liver lysate from BMAL1-deficeient mouse. CIP, calf intestine alkaline phosphatase; arrows, BMAL1; *, a phosphorylated band. [file 1471-2199-9-1-S3.tiff]

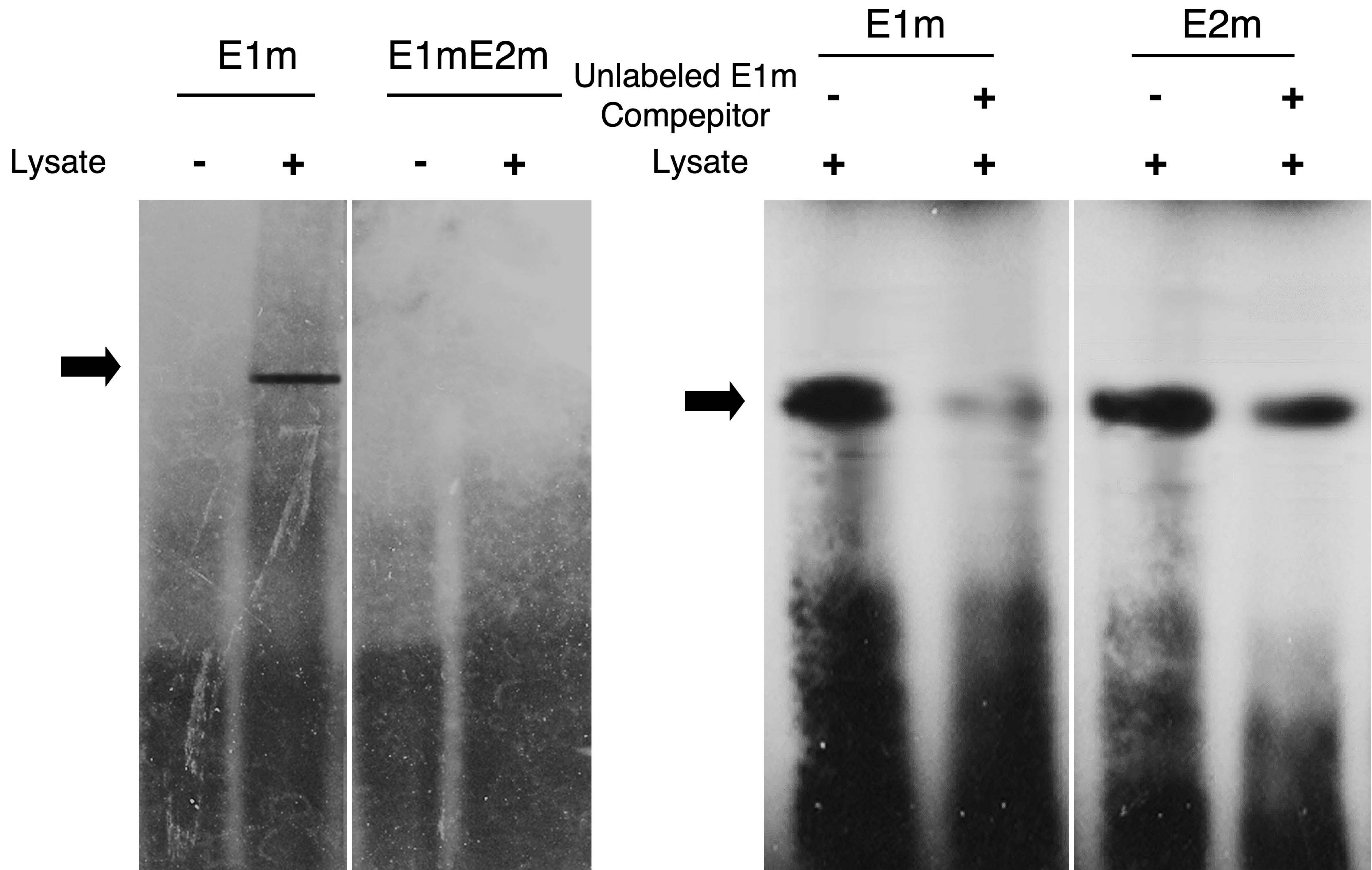

Supplement: Additional file 5 — Some protein may bind to E2. Electrophoretic mobility shift assay (EMSA) shows that some protein from cellular lysates binds to E2 (left). The bands from liver lysates in E1m were attenuated with excess of E1m, but not in E2m (right). [file 1471-2199-9-1-S5.pdf]
