# Supplementary material for: A direct repeat of E-box-like elements is required for cell-autonomous circadian rhythm of clock genes
Source: BMC Mol Biol. 2008 Jan 4;9:1. doi: 10.1186/1471-2199-9-1 (PMC2254435; doi:10.1186/1471-2199-9-1)
Supplement: Additional file 4 — The EE-element of hDbp promoter is sufficient for circadian rhythm generation. (A) Wild-type and mutant sequence of hDbp E1 and E2 and their flanking region are shown. The core sequence within the EE-element is shown in capital letters. (B) EE-element-driven luciferase bioluminescence by IV-ROMS. Gray lines indicate bioluminescence of wild type hDbp EE-element; and black lines show those of mutants. The abscissa presents "day"; and the ordinate shows "relative luciferase intensity". First peak values of the curves were set to 1. (C) Wild-type (sp6; gray letters) and mutant (sp4, 5, 7, 8; solid letters) sequences of hDBP EE-element and their flanking sequences are shown. Underlines indicate inserted nucleotides into space of the EE-element. The core sequence within the EE-element is shown in capital letters. (D) EE-element-driven luciferase bioluminescence by IV-ROMS. Black lines indicate bioluminescence of the mutant hDbp EE-elements; and the gray lines show those of wild type. The abscissa presents "day"; and the ordinate shows "relative luciferase intensity". First peak values of the curves were set to 1. [file 1471-2199-9-1-S4.pdf]

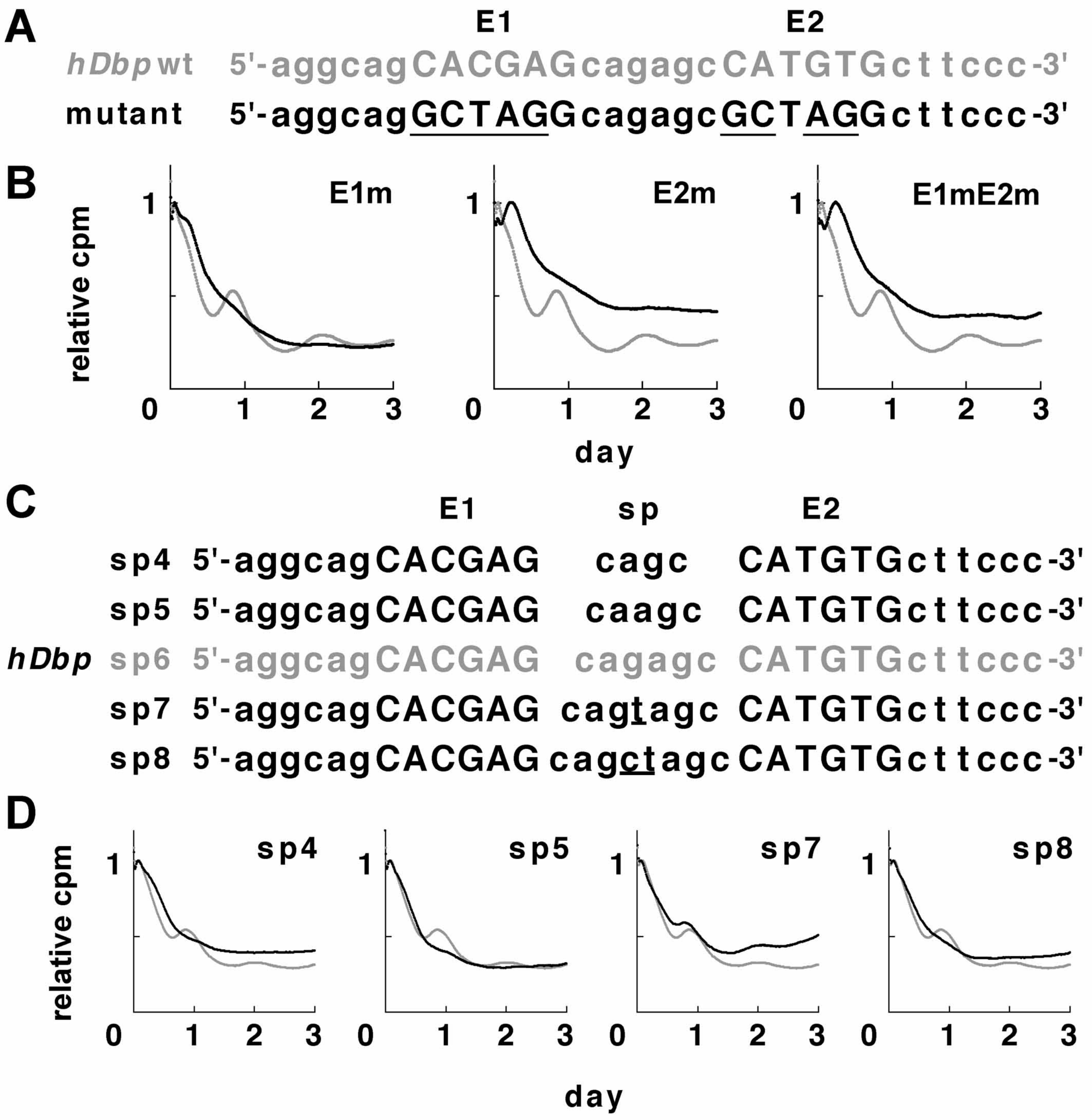
A
E1
E2
hDbp wt 5'-aggcagCACGAGcagagcCATGTGcttccc-3'
mutant 5'-aggcagGCTAGGcagagcGCTAGGcttccc-3'
B
relative cpm
1
E1m
E2m
E1mE2m
0 1 2 3
day
C
E1
sp
E2
sp4 5'-aggcagCACGAG cagc CATGTGcttccc-3'
sp5 5'-aggcagCACGAG caagc CATGTGcttccc-3'
hDbp sp6 5'-aggcagCACGAG cagagc CATGTGcttccc-3'
sp7 5'-aggcagCACGAG cagtagc CATGTGcttccc-3'
sp8 5'-aggcagCACGAGcagctagcCATGTGcttccc-3'
D
relative cpm
1
sp4
sp5
sp7
sp8
0 1 2 3
day
