# Supplementary material for: A direct repeat of E-box-like elements is required for cell-autonomous circadian rhythm of clock genes
Source: BMC Mol Biol. 2008 Jan 4;9:1. doi: 10.1186/1471-2199-9-1 (PMC2254435; doi:10.1186/1471-2199-9-1)
Supplement: Additional file 6 — The information of constructs and genes used in this study. Upper panel shows the construct sequences used in this study. Middle panel demonstrates the oligonucleotides used in Figure 2C. Bottom panel shows the accession numbers of Pers and Dbp used. [file 1471-2199-9-1-S6.pdf]

A

IV-ROMS		SacI		E1	Space	E2		NheI	
hPer1 wt	5'-	C	CAGGTC	CACGTG	CGCCCG	TGTGTG	TGACAC	G	-3'
	3'-	TCGAG	GTCCAG	GTGCAC	GCGGGC	ACACAC	ACTGTG	CGATC	-5'
hPer2 wt	5'-	C	CGCGGT	CACGTT	TTCCAC	TATGTG	ACAGCG	G	-3'
	3'-	TCGAG	GCGCCA	GTGCAA	AAGGTG	ATACAC	TGTCGC	CGATC	-5'
hPer3 wt	5'-	C	GACCGG	CACGCG	GCGAGC	CTCGAG	ACTGCG	G	-3'
	3'-	TCGAG	CTGGCC	GTGCGC	CGCTCG	GAGCTC	TGACGC	CGATC	-5'
hPer3 GTGm	5'-	C	GACCGG	CACGCG	GCGAGC	CTCGTG	ACTGCG	G	-3'
	3'-	TCGAG	CTGGCC	GTGCGC	CGCTCG	GAGCAC	TGACGC	CGATC	-5'
hPer2 E1m	5'-	C	CGCGGT	GCTAGT	TTCCAC	TATGTG	ACAGCG	G	-3'
	3'-	TCGAG	GCGCCA	CGATCA	AAGGTG	ATACAC	TGTCGC	CGATC	-5'
hPer2 E2m	5'-	C	CGCGGT	CACGTT	TTCCAC	GCTAGG	ACAGCG	G	-3'
	3'-	TCGAG	GCGCCA	GTGCAA	AAGGTG	CGATCC	TGTCGC	CGATC	-5'
hPer2 E1mE2m	5'-	C	CGCGGT	GCTAGT	TTCCAC	GCTAGG	ACAGCG	G	-3'
	3'-	TCGAG	GCGCCA	CGATCA	AAGGTG	CGATCC	TGTCGC	CGATC	-5'
hPer2 sp4	5'-	C	CGCGGT	CACGTT	TTAC	TATGTG	ACAGCG	G	-3'
	3'-	TCGAG	GCGCCA	GTGCAA	AATG	ATACAC	TGTCGC	CGATC	-5'
hPer2 sp5	5'-	C	CGCGGT	CACGTT	TTCAC	TATGTG	ACAGCG	G	-3'
	3'-	TCGAG	GCGCCA	GTGCAA	AAGTG	ATACAC	TGTCGC	CGATC	-5'
hPer2 sp7	5'-	C	CGCGGT	CACGTT	TTCTCAC	TATGTG	ACAGCG	G	-3'
	3'-	TCGAG	GCGCCA	GTGCAA	AAGAGTG	ATACAC	TGTCGC	CGATC	-5'
hPer2 sp8	5'-	C	CGCGGT	CACGTT	TTCCTCAC	TATGTG	ACAGCG	G	-3'
	3'-	TCGAG	GCGCCA	GTGCAA	AAGGAGTG	ATACAC	TGTCGC	CGATC	-5'
hDbp wt	5'-	C	AGGCAG	CACGAG	CAGAGC	CATGTG	CTTCCC	G	-3'
	3'-	TCGAG	TCCGTC	GTGCTC	GTCTCG	GTACAC	GAAGGG	CGATC	-5'
hDbp E1m	5'-	C	AGGCAG	GCTAGG	CAGAGC	CATGTG	CTTCCC	G	-3'
	3'-	TCGAG	TCCGTC	CGATCC	GTCTCG	GTACAC	GAAGGG	CGATC	-5'
hDbp E2m	5'-	C	AGGCAG	CACGAG	CAGAGC	GCTAGG	CTTCCC	G	-3'
	3'-	TCGAG	TCCGTC	GTGCTC	GTCTCG	CGATCC	GAAGGG	CGATC	-5'
hDbp E1E2m	5'-	C	AGGCAG	GCTAGG	CAGAGC	GCTAGG	CTTCCC	G	-3'
	3'-	TCGAG	TCCGTC	CGATCC	GTCTCG	CGATCC	GAAGGG	CGATC	-5'

B

Oligo Pull-down				E1	Space	E2			
hPer2 wt	5'-	GCGCG	CGCGGT	CACGTT	TTCCAC	TATGTG	ACAGCG	GAGGG	-3'
	3'-	CGCGC	GCGCCA	GTGCAA	AAGGTG	ATACAC	TGTCGC	CTCCC	-5'
hPer2 E1m	5'-	GCGCG	CGCGGT	GCTAGG	TTCCAC	TATGTG	ACAGCG	GAGGG	-3'
	3'-	CGCGC	GCGCCA	CGATCC	AAGGTG	ATACAC	TGTCGC	CTCCC	-5'
hPer2 E2m	5'-	GCGCG	CGCGGT	CACGTT	TTCCAC	GCTAGG	ACAGCG	GAGGG	-3'
	3'-	CGCGC	GCGCCA	GTGCAA	AAGGTG	CGATCC	TGTCGC	CTCCC	-5'

C

species	genome version	accession number			
		Per1	*Per2*	*Per3*	*Dbp*
human	Mar. 2006	NM_002616	NM_022817	NM_016831	NM_001352
rhesus	Jan. 2006	NM_002616 (not rhesus)	NM_022817 (not rhesus)	NM_016831 (not rhesus)	
rat	Nov. 2004	NM_001034125	NM_031678	NM_023978	
mouse	Feb. 2006	NM_011065	NM_011066	NM_011067	
opossum	Jan. 2006	NM_011065 (not opossum)	NM_011066 (not opossum)	NM_011067 (not opossum)	
